# Dynamic DNA Methylation During Aging: A “Prophet” of Age-Related Outcomes

**DOI:** 10.3389/fgene.2019.00107

**Published:** 2019-02-18

**Authors:** Fu-Hui Xiao, Hao-Tian Wang, Qing-Peng Kong

**Affiliations:** ^1^State Key Laboratory of Genetic Resources and Evolution, Kunming Institute of Zoology, Chinese Academy of Sciences, Kunming, China; ^2^Center for Excellence in Animal Evolution and Genetics, Chinese Academy of Sciences, Kunming, China; ^3^Key Laboratory of Healthy Aging Research of Yunnan Province, Kunming, China; ^4^Kunming Key Laboratory of Healthy Aging Study, Kunming, China; ^5^KIZ/CUHK Joint Laboratory of Bioresources and Molecular Research in Common Diseases, Kunming, China; ^6^Kunming College of Life Science, University of Chinese Academy of Sciences, Beijing, China

**Keywords:** DNA methylation, epigenetic clock, age, age-related outcome, prediction

## Abstract

The biological markers of aging used to predict physical health status in older people are of great interest. Telomere shortening, which occurs during the process of cell replication, was initially considered a promising biomarker for the prediction of age and age-related outcomes (e.g., diseases, longevity). However, the high instability in detection and low correlation with age-related outcomes limit the extension of telomere length to the field of prediction. Currently, a growing number of studies have shown that dynamic DNA methylation throughout human lifetime exhibits strong correlation with age and age-related outcomes. Indeed, many researchers have built age prediction models with high accuracy based on age-dependent methylation changes in certain CpG loci. For now, DNA methylation based on epigenetic clocks, namely epigenetic or DNA methylation age, serves as a new standard to track chronological age and predict biological age. Measures of age acceleration (Δage, DNA methylation age – chronological age) have been developed to assess the health status of a person. In addition, there is evidence that an accelerated epigenetic age exists in patients with certain age-related diseases (e.g., Alzheimer’s disease, cardiovascular disease). In this review, we provide an overview of the dynamic signatures of DNA methylation during aging and emphasize its practical utility in the prediction of various age-related outcomes.

## Introduction

Aging is an inevitable biological process accompanied by progressive decline in physical function and increased risk of multiple age-related diseases, such as cardiovascular disease, neurodegenerative disease, and cancer (Sen et al., 2016). Currently, human populations across the world are rapidly aging (Lutz et al., 2008; Christensen et al., 2009). Age-related chronic diseases account for most global diseases as well as morbidity and mortality (Kennedy et al., 2014). For example, cardiovascular disease, one of the most common diseases of aging, accounted for 30% of all deaths worldwide per year (Pagidipati and Gaziano, 2013; Nichols et al., 2014). Consequently, the development of tools to diagnose and predict age-dependent risks has enormous significance in preventing age-related diseases and improving the health status of the elderly.

The process of aging results in multiple changes at both the molecular and cellular level, including cellular senescence, telomere attrition, and epigenetic alterations (Lopezotin et al., 2013). Among these hallmarks, telomere length, which experiences progressive shortening during replication of somatic cells, is a remarkable characteristic of aging and linked with age-related health status (Rizvi et al., 2015). However, recent evidence has revealed that the correlation between telomere length and age-related outcomes of individuals is low (Müezzinler et al., 2013; Breitling et al., 2016; Marioni et al., 2016). Thus, investigators are still searching for other biomarkers that can be used in the prediction of age-related outcomes with higher accuracy.

Current studies have indicated that epigenetic changes comprise a significant component of the aging process (Jones et al., 2015). Epigenetics refer to the modulation of gene activity without any change in the genomic sequence. Well-studied epigenetic modifications include DNA methylation, histone modification, and non-coding RNA, with changes in dynamic DNA methylation found to be most associated with the aging process (Richardson, 2003b; Fraga and Esteller, 2007; Sen et al., 2016). In general, age-dependent changes in DNA methylation include global hypomethylation and region-specific hypermethylation (Xiao et al., 2016). Abundant studies have demonstrated a close relationship between DNA methylation and aging and longevity (Feinberg et al., 2002; Robertson, 2005; Xiao et al., 2016). These findings have impelled researchers to develop age predictors based on the correlation between methylation changes and chronological age (Hannum et al., 2013; Horvath, 2013). DNA methylation age, evaluated by these predictors, reflects the biological age of a person, which has a close association with individuals’ health status and can be changed by multiple risk factors, such as smoking and obesity (Dugué et al., 2018). Therefore, the difference between DNA methylation age and chronological age (i.e., Δage) may be a promising tool in predicting disease risk and longevity potential in early life (Vaiserman, 2018). Here, we review the dynamics of methylation in aging and discuss the roles of age-dependent methylation changes in the prediction of age and age-related outcomes.

## Dna Methylation

DNA methylation, a well-studied epigenetic modification, refers to the transfer of a methyl (CH3) group from *S*-adenosyl methionine (SAM) to the fifth position of cytosine nucleotides, forming 5-methylcytosine (5mC) (Hotchkiss, 1948; Chiang et al., 1996; Moore et al., 2013). This process is catalyzed by at least three DNA methyltransferases, including Dnmt1, Dnmt3a, and Dnmt3b (Okano et al., 1999; Vilkaitis et al., 2005). Among these enzymes, Dnmt1 is responsible for the maintenance of methylation patterns in the genome by replicating the hemimethylated CpG sites (Vilkaitis et al., 2005), whereas Dnmt3a/b are considered *de novo* methyltransferases (Okano et al., 1999). Otherwise, evidence has shown that DNA demethylation can be achieved by either passive or active mechanism (Chen and Riggs, 2011). The passive demethylation can be caused by the inhibition of Dnmt1 during cell replication (Wolffe et al., 1999); while the active demethylation is modulated by the DNA demethylases. In the past, 5mC DNA glycosylase (5-MCDG) and methyl-CpG binding domain protein 4 (MBD4) have been served with the activity of DNA demethylase (Jost et al., 1999; Hendrich et al., 1999; Zhu, 2009). Recent years, amounting evidence has shown ten-eleven translocation (TET) dioxygenases play important roles in DNA demethylation through converting 5-methylcytosine to 5-hydroxymethylcytosine (Jin et al., 2014; Ichiyama et al., 2015).

In mammalian cells, most 5mC occurs at nucleic sequences in the context of cytosine-phosphate-guanine (CpG) dinucleotides. About 70–80% of CpG sites are methylated in human somatic cells, with most unmethylated CpG sites clustered in the CpG island located on the promoter region of the genes (Ehrlich et al., 1982; Lister et al., 2009). Accumulated evidence has shown that DNA methylation plays essential roles in many biological processes, including gene regulation, chromosome stability, genomic imprinting, and X chromosome inactivation (Robertson, 2005). Many studies have revealed that mammalian developmental processes cannot depart from modulation of DNA methylation (Trowbridge et al., 2009). One remarkable case comes from stem-cell differentiation. All myeloid and lymphoid blood lineages are differentiated from hematopoietic stem cells (HSCs) (Chao et al., 2008), during which the activity of genes (e.g., *KCNH2, SUSD3*) that control cell fate is highly regulated by methylation status (Farlik et al., 2016). Conversely, abnormal DNA methylation is related to the occurrence of several human diseases (Feinberg et al., 2002; Robertson, 2005), with many studies showing the role of aberrant DNA methylation in the activation of tumor promoter genes (e.g., *MAGE, S100P*) and silencing of tumor suppressor genes (e.g., *VHL, MLH1*) in various cancers (Herman and Baylin, 2003; Sato et al., 2004). Additionally, there are researches that abnormal methylation plays an important role in the pathogenesis of autoimmune diseases (e.g., idiopathic human lupus), metabolic syndromes (e.g., hyperglycemia), and neurological disorders (e.g., autism spectrum disorder) (Richardson 2003a; Côté et al., 2016; Andrews et al., 2018).

Generally, DNA methylation in regions near the transcription start site (TSS) is closely associated the suppression of gene expression (Bird, 1986; Eden and Cedar, 1994). Accumulating evidence has shown that transcriptional suppression of DNA methylation involves prevention of transcription activation factor (e.g., AP-2) or recruitment of transcription inhibiting factor (e.g., MeCP2) binding to TSS regions (Comb and Goodman, 1990; Jones et al., 1998; Fuks et al., 2003). On the contrary, there are reports that gene body methylation likely increases transcriptional activity (Maunakea et al., 2010; Yang et al., 2014). Emerging evidence has shown that DNA methylation on gene body functions can protect the gene body from spurious transcripts by guaranteeing the fidelity of mRNA transcription initiation (Neri et al., 2017).

## Association Between Dynamic Dna Methylation and Aging/Age-Related Diseases

**FIGURE 1:**
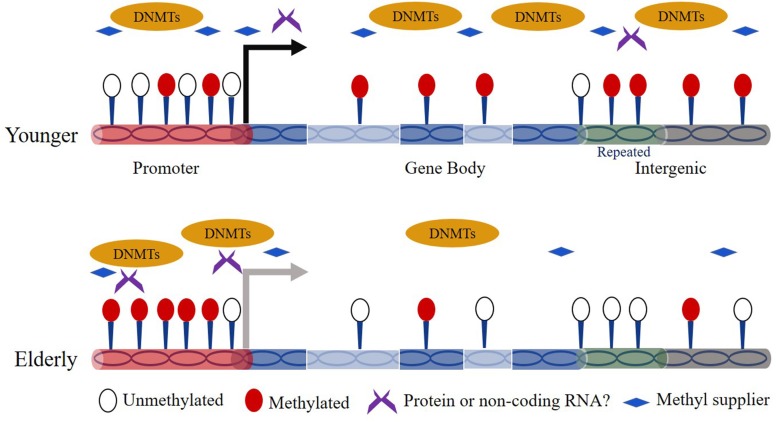
Overview of mechanisms of dynamic DNA methylation during aging.

The association between DNA methylation and aging has been studied for decades. Thirty-five years ago, Wilson and Jones (1983) observed a marked decrease in 5mC content in aged normal diploid fibroblasts of mice, hamsters, and humans. Since then, age-dependent genome-wide DNA hypomethylation especially on interspersed repetitive sequence (IRS) has been detected in a variety of cell types from different tissues and organs (e.g., blood, brain) (Vanyushin et al., 1973; Bollati et al., 2009; Jintaridth and Mutirangura, 2010). Over the human lifetime, the content of 5mC is highest in embryos and decreases gradually as individuals age (Goel et al., 2017). In addition, region/site-specific hypermethylation is also broadly observed in the genome during aging. For example, Rakyan et al. (2010) revealed that hypermethylated regions in human aging are preferentially located on bivalent chromatin domains and Oakes et al. (2003) showed that ribosomal DNA in the genome exhibits increased methylation in aged rats. For the time being, evidence has shown that the global reduced methylation content can be caused by down-regulated expression of Dnmts or insufficient supply of folic acid in elderly subjects (Figure 1) (Rampersaud et al., 2000; Ciccarone et al., 2016). Other works revealed that the risk factors like UV-B light, air pollution, and smoke facilitate the global hypomethylation (Prins et al., 2013; Puttipanyalears et al., 2013; Wu et al., 2013). In addition, Fernández et al. (2015) revealed that the hypomethylated sites preferentially occurs at H3K4me1-rich regions. However, the studies on the mechanism of site-specific hypermethylation currently remain very limited. Nevertheless, one hypothesis indicates that Dnmts can target specific genomic sequences based on a RNA interference mechanism (Morris et al., 2004). Moreover, there is report that the genomic regions bound by the Polycomb complex tend to be hypermethylated during aging (Jung and Pfeifer, 2015).

The crucial function of DNA methylation in multiple key processes has driven researchers to focus on the contribution of dynamic methylation changes to age-related diseases. In accordance with age-dependent hypomethylation, several age-related diseases, including neurodegenerative disease, cardiovascular disease, and cancer, show close association with marked global methylation decrease (Ehrlich, 2009; Baccarelli et al., 2010; Chouliaras et al., 2013). In addition, the examination of abnormal methylation events in certain genes provides direct evidence to strengthen the close relationship between DNA methylation and disease expression. For instance, Ozaki et al. (2017) revealed that the loss of methylation in three CpG loci in intron 1 of *TREM2*, an Alzheimer’s disease susceptibility gene, results in higher expression of *TREM2* in the leukocytes of Alzheimer’s disease subjects. Ling et al. (2008) showed that *PPARGC1A*, a gene with effects on insulin secretion, exhibits significantly lower expression in islets from patients with type 2 diabetes and the down-regulation of *PPARGC1A* mRNA is caused by the increase in methylation of its promoter.

In addition, dynamic epigenetic changes during lifetimes serve as an important mechanism for organisms to adapt the external and internal environmental changes (D’Aquila et al., 2013; Schrey et al., 2016). Therefore, some dynamic methylation events during aging likely function as beneficial adaptive changes to response the stress exposure throughout the life-course. For instance, there is case that individuals can retain a high level of glucose across the famine period through methylation-based inhibition of *IGF2* expression (Heijmans et al., 2008). Nevertheless, further studies are required to determine the age-related CpG sites with beneficial effects that are common across individuals.

## Dna Methylation-Based Age Prediction

Growing evidence has demonstrated the successful utilization of epigenetic biomarkers in predicting age with high accuracy (Li et al., 2013; Goel et al., 2017). Researchers have recently developed multiple age-prediction models with various statistical methods to determine the age of a person based on the age-dependent methylation changes in certain CpG loci (Bocklandt et al., 2011; Hannum et al., 2013; Horvath, 2013; Weidner et al., 2014). The number of CpG sites used in building these age predication models ranges from several to 100s. Effort has also been expended to increase the practicability of age predictors and the use of as few loci as possible. For example, Bocklandt et al. (2011) built an age-prediction model using just two CpG sites with a linear relationship between methylation and age in the saliva of twins and obtained an average accuracy of 5.2 years. Weidner et al. (2014) developed an age-prediction model with three CpG sites that showed age-dependent methylation changes in human blood cells, with an accuracy of less than 5 years. Others have focused on improving the stability and accuracy of the tools and utilized many CpG sites. Two well-known age predictors (epigenetic clocks) include Hannum’s clock and Horvath’s clock, which contain 71 and 353 CpG sites, respectively (Hannum et al., 2013; Horvath, 2013), and show enhanced accuracy of 3–4 years. Notably, Hannum’s clock is uniquely suitable for human blood, whereas Horvath’s clock is appropriate for multiple human tissues and cells.

## Dna Methylation Age Acceleration Predicts the Risk of Age-Related Disorders

Initially, the practicability of DNA methylation-based age predictors was considered useful in the field of forensic genetics (Yi et al., 2015). However, researchers have now proposed that DNA methylation age can also reflect the biological age, not just the chronological age, of a person (Weidner et al., 2014; Chen et al., 2016; Zhang et al., 2017a). The concept of biological age is to explain the variation in the biological status of individuals with the same chronological age (Andrews et al., 2017). Indeed, there is increasing evidence that the difference between biological age (i.e., DNA methylation age) and chronological age, often symbolized as Δage (DNA methylation age – chronological age), is closely associated with age-related disorders. Several research groups have observed an acceleration in DNA methylation age in some age-associated diseases, including Alzheimer’s disease, cardiovascular disease, and cancer (Perna et al., 2016; McCartney et al., 2018). Other cases also support the association of DNA methylation age acceleration in the expression of certain disorders. For instance, Davis et al. (2017) observed a correlation between accelerated DNA methylation age and elevated diurnal cortisol production, which is closely linked with a reduction in hippocampal volume. Marioni et al. (2015b) showed that greater DNA methylation acceleration is correlated with lower cognitive score, weaker grip strength, and poorer lung function in humans during later life. Horvath et al. (2015a) found accelerated DNA methylation age in Down syndrome patients with clinical signatures of “accelerated aging.” There is also evidence that frailty, a syndrome with a pronounced association with age-related phenotypes, has a significant association with DNA methylation age but not with telomere length (Breitling et al., 2016). Moreover, amounting evidence has shown that the Δage is able to predict the mortality in the later life (Marioni et al., 2015a; Zheng et al., 2016). For example, Marioni et al. (2015a) revealed that a 5-year higher Hannum and Horvath Δage are associated with a 21 and 11% of greater mortality, respectively. Zheng et al. (2016) pointed out that DNA methylation age estimated from blood tissue can also be used to predict cancer incidence and mortality. However, another study of Zhang et al. (2017b) analyzed the CpG sites that are specific to the indicative of disease-related outcomes and mortality and showed a big difference with those in tracking natural aging. Accordingly, we suppose that it is necessary to develop directed prediction models for various age-related outcomes through integrating the age-related and disease-specific CpG sites.

The intrinsic and extrinsic factors contributing to the acceleration of epigenetic age have also attracted attention. One broadly focused risk factor is cumulative lifetime stress, with its contribution to accelerated DNA methylation age revealed in many studies (Zannas et al., 2015; Wolf et al., 2016, 2018a,b). In addition, the finding that certain CpG loci affected by drinking and smoking present the same methylation changes as aging suggests the potential roles of these activities in accelerating DNA methylation age (Xiao et al., 2018). Other factors include age at menopause, which is closely associated with epigenetic age acceleration in women (Levine et al., 2016). A correlation between obesity and accelerated epigenetic aging has also been observed in middle-aged individuals (Nevalainen et al., 2017).

## Relationship Between Dna Methylation Age and Longevity

Longevity, which is a trait of extreme aging, is also associated with age-related DNA methylation changes (Gravina and Vijg, 2010; Jones et al., 2015; Xiao et al., 2016). A growing body of research has investigated the methylation signatures of long-lived people (e.g., centenarians) (Heyn et al., 2012; Gentilini et al., 2013; Xiao et al., 2015), who exhibit a delayed age of onset for some age-associated diseases (Hitt et al., 1999; Engberg et al., 2009; Andersen et al., 2012). Previous studies indicate that the specific methylation profiles of long-lived people have potential roles in suppressing the occurrence of age-related diseases (Xiao et al., 2015). In addition, research has shown that semi-supercentenarians and their offspring have a relatively younger biological age as reflected by their decreased DNA methylation age (Horvath et al., 2015b). What’s more, Lin et al. (2016) suggested that DNA methylation level of several CpG sites (e.g., associated with *PDE4C* and *CLCN6*) likely has an association with life-expectancy. Interestingly, there are emerging reports that the epigenetic clocks tick faster in mice than those in humans, indicating a potential association between epigenetic clocks and the maximum life-span of mammals (Stubbs et al., 2017; Wagner, 2017; Lowe et al., 2018).

## Conclusion and Perspectives

**FIGURE 2:**
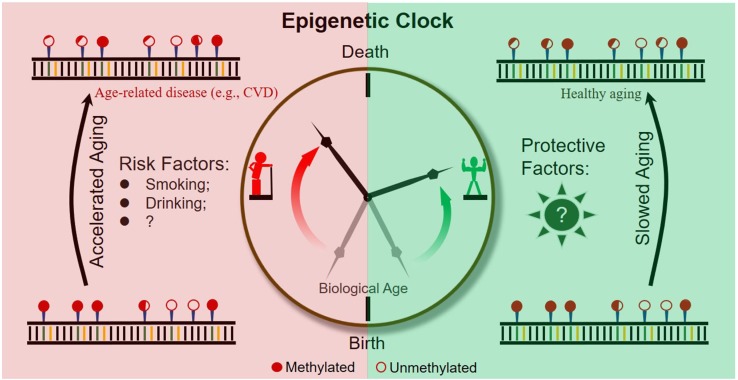
Schematic diagram of an epigenetic clock across a human lifetime.

It is becoming evident that epigenetic clocks based on the dynamic methylation of certain CpG loci during aging are of help in the prediction of chronological and biological age (Figure 2). In addition, abundant research indicates that the ticking rate of an epigenetic clock is associated with age-related diseases and longevity. Taken together, evidence obtained so far suggests the great potential of dynamic methylation as a “prophet” of age-related outcomes, including the pathology and health status of a person.

However, to further expand the practical application of epigenetic clocks, researchers should make efforts to address the following two important issues. First, as current epigenetic clocks are only correlated with age-related outcomes, the development of specific epigenetic clocks by combining outcome-specific CpG sites is necessary. Second, to slow down the rate of epigenetic aging throughout the lifetime of humans, risk factors accelerating epigenetic clocks and protective factors slowing epigenetic clocks should be identified and extensively studied (Figure 2).

## Ethics Statement

The research protocol was approved by the Ethics Committee at Kunming Institute of Zoology, Chinese Academy of Sciences.

## Author Contributions

Q-PK and F-HX conceived the project and wrote the manuscript. H-TW revised the manuscript.

## Conflict of Interest Statement

The authors declare that the research was conducted in the absence of any commercial or financial relationships that could be construed as a potential conflict of interest.
